# High-intensity interval training: a review of its impact on glucose control and cardiometabolic health

**DOI:** 10.1007/s00125-016-4106-1

**Published:** 2016-09-28

**Authors:** Sophie Cassidy, Christian Thoma, David Houghton, Michael I. Trenell

**Affiliations:** 1 MoveLab, Institute of Cellular Medicine, The Medical School, Newcastle University, 4th Floor William Leech Building, Framlington Place, Newcastle upon Tyne, NE2 4HH UK; 2 School of Interprofessional Health Studies, Auckland University of Technology, Auckland, New Zealand

**Keywords:** Cardiovascular system, Exercise, Exercise therapy, Metabolic diseases, Metabolism, Physical fitness, Review, Weight loss

## Abstract

Exercise plays a central role in the management and treatment of common metabolic diseases, but modern society presents many barriers to exercise. Over the past decade there has been considerable interest surrounding high-intensity interval training (HIIT), with advocates claiming it can induce health benefits of similar, if not superior magnitude to moderate-intensity continuous exercise, despite reduced time commitment. As the safety of HIIT becomes clearer, focus has shifted away from using HIIT in healthy individuals towards using this form of training in clinical populations. The continued growth of metabolic disease and reduced physical activity presents a global health challenge and effective therapies are urgently required. The aim of this review is to explore whether the acclaim surrounding HIIT is justified by examining the effect of HIIT on glucose control, its ability to affect cardiovascular function and the underlying mechanisms of the changes observed in those with common metabolic diseases. It also explores translation of the research into clinical practice.

## Why exercise?

Before the agricultural, industrial and digital ages, humans expended large amounts of energy in activities centred on maintaining shelter and procuring food and water [1]. Fast forward some 350 generations and the barriers to exercise and physical activity in the 21st century are enormous. Sedentary behaviours, such as the use of mechanised transport and screen-based leisure pursuits have become the norm in modern society. There is an urgent need therefore to find practical, attractive and effective exercise therapies to combat the wave of inactivity sweeping through the western world.

Not only is exercise part of our nature, it is strongly associated with reduced chronic disease risk. Globally, metabolic disorders such as the metabolic syndrome, non-alcoholic fatty liver disease (NAFLD), type 2 diabetes and the closely associated cluster of cardiovascular diseases are rapidly increasing [2]. European and US treatment algorithms for these obesity driven epidemics recommend weight loss and maintenance as a main priority across all stages [3, 4]. Conceivably, this can be achieved through energy restriction and/or physical exercise.

Current management guidelines for these common metabolic conditions advise individuals to undertake around 150 min of moderate-to-vigorous aerobic exercise per week, spread over most days of the week, in addition to resistance training on at least 2 days of the week [5, 6]. The emphasis remains on moderate-intensity continuous training (MICT); however there is mounting evidence that high-intensity interval training (HIIT) provides an alternative means of achieving the same or greater health benefits vs MICT, provided there are no medical contraindications to engaging in HIIT and that it is well tolerated and preferred by the individual taking part. We refer readers to recent meta-analyses for a comprehensive analysis of the metabolic [7] and cardiorespiratory [8] benefits of HIIT in patient groups. The aim of this review is to assimilate existing evidence and provide a clinically relevant narrative of the cardiometabolic benefits of HIIT in those with common metabolic diseases, before moving onto discussing its safety profile, tolerability and practical considerations for translation into clinical care. The information presented in this review is not part of a formal systematic review and, therefore, may not have been subjected to the rigor required for such a summary of the data currently available on HIIT.

## What is HIIT?

**Fig. 1:**
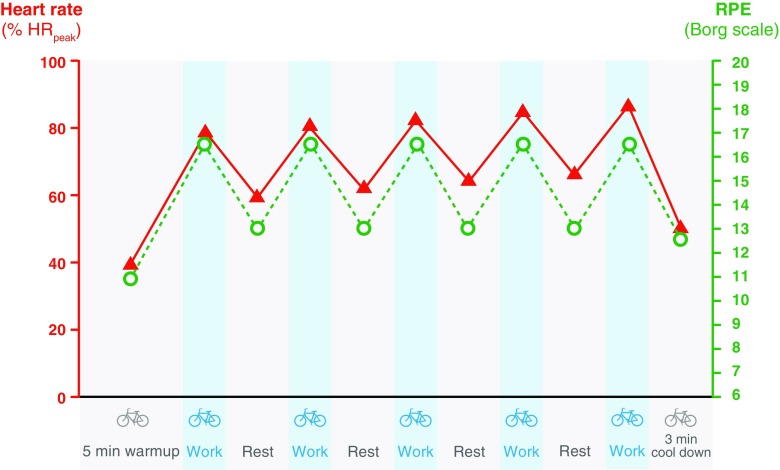
An example of a HIIT protocol. Schematic of the HIIT protocol adopted by our group in adults with NAFLD [39] and type 2 diabetes [38]. Intensity was based upon the perceived rate of exertion (RPE), inducing a strong acute physiological response in heart rate (shown as % peak heart rate [HR_peak_]), which increases across intervals

HIIT can be described as ‘brief intervals of vigorous activity interspersed with periods of low activity or rest’, which induces a strong acute physiological response (Fig. 1) [9]. A number of HIIT protocols have been adopted in the literature (see Table 1), but the majority of interventions use high-intensity intervals lasting between 1 and 4 min. The goal of HIIT is to accumulate activity at an intensity that the participant would be unable to sustain for prolonged periods (i.e. 80–95% of peak oxygen consumption ($\overset{.}{V}{\mathrm{O}}_{2\mathrm{peak}}$) or >90% of maximum heart rate (HR_max_), therefore the recovery time should be sufficient to allow the subsequent interval to be completed at the desired intensity. The total duration of a HIIT session tends to be ≥20 min, which actually makes it comparable with recommendations for MICT, in terms of duration. There is also a sub-category of HIIT involving 10–30 second intervals and intensities often exceeding 100% $\overset{.}{V}{\mathrm{O}}_{2\mathrm{peak}}$, i.e. ‘all-out’ exercise at a workload that is above maximal aerobic capacity [10]. This is often called sprint interval training and has not been substantially tested in clinical populations and will therefore not be covered further.

**Table 1:**
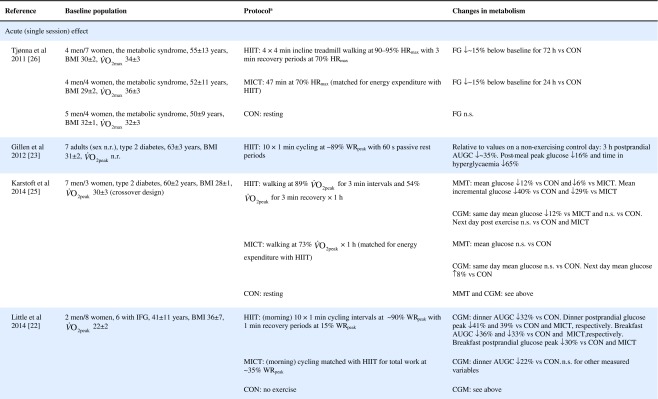

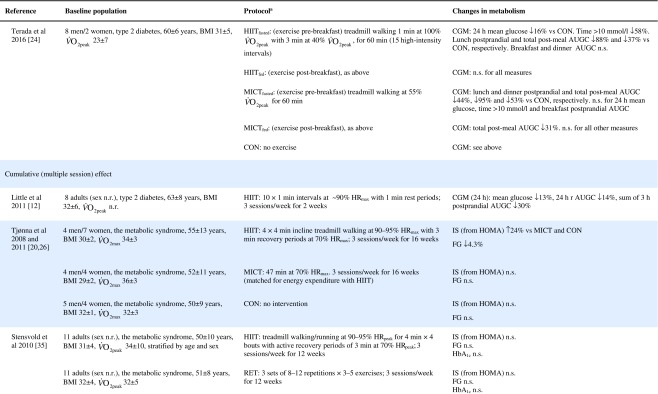

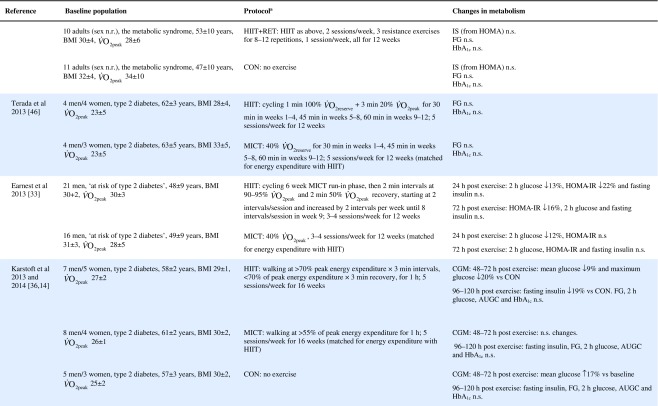

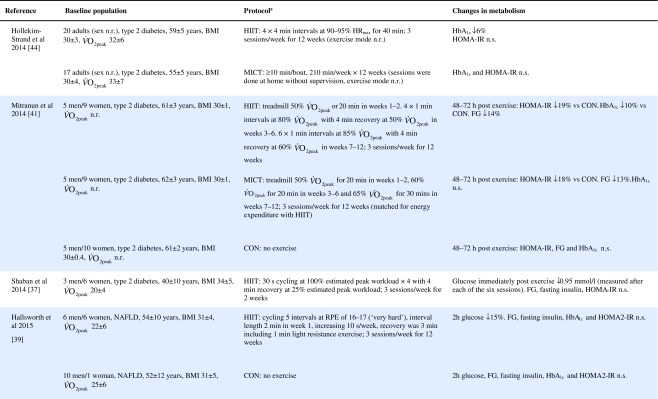

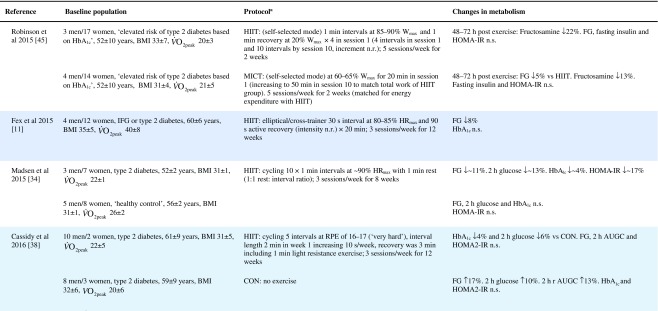
Effect of HIIT on insulin and glucose metabolism in patients with the metabolic syndrome, NAFLD or type 2 diabetes

The vast majority of the published HIIT research, particularly in clinical populations, has used exercise modalities involving cycling, walking, and running, mostly carried out on stationary cycles and treadmills (see Table 1). However, other equipment, such as cross-trainers/ellipiticals [11], are reasonable options for some. Evidently there is clear variation throughout the literature and it still remains to be determined whether an optimal HIIT protocol exists for metabolic disease management.

## HIIT and metabolic health

### Skeletal muscle molecular adaptations

**Fig. 2:**
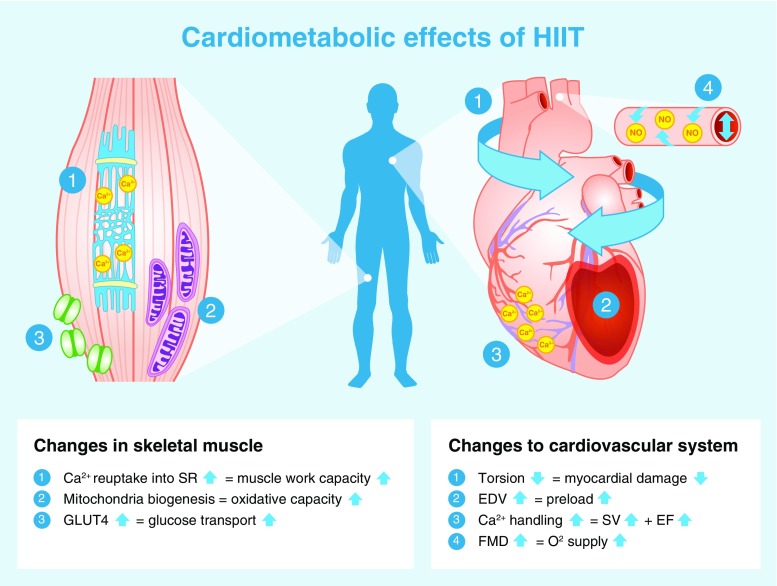
Cardiometabolic effects of HIIT. The figure depicts the previously reported muscular and cardiovascular impact of HIIT in those with common metabolic diseases. In boxes of text: upward arrow, increase; downward arrow, decrease. EDV, end diastolic volume; EF, ejection fraction; FMD, flow mediated dilation; SR, sarcoplasmic reticulum; SV, stroke volume

A number of molecular adaptations have been identified within skeletal muscle following HIIT (Fig. 2). Skeletal muscle is the primary site for glucose disposal via insulin- and non-insulin-mediated glucose uptake; the latter stimulated by muscular contraction. It therefore plays a large role in regulating metabolism.

#### Increased GLUT-4 content

GLUT-4 content in the vastus lateralis increased by 369% following six sessions of HIIT in type 2 diabetes patients [12]. Insulin resistance underlies metabolic disease and although decreased GLUT-4 content is not the cause of insulin resistance, any increase in this protein improves glucose transport within skeletal muscle [13]. Another study found that 16 weeks of HIIT in type 2 diabetes patients induced higher membrane-bound GLUT-4 and *GLUT-4* mRNA levels in comparison with energy matched MICT, but no rise in overall GLUT-4 protein content was observed [14]. The reduced intensity of intervals in this study (70% $\overset{.}{V}{\mathrm{O}}_{2\mathrm{peak}}$) compared with other HIIT protocols is worth noting, however, as is the fact that biopsies were obtained 5–6 days post-training.

#### Mitochondrial adaptations

Reduced mitochondrial content [15], mitochondrial function [16] and markers of mitochondrial biogenesis in skeletal muscle are commonly observed in individuals with metabolic disease [17] and have been suggested to contribute to insulin resistance. In adults with type 2 diabetes, 2 weeks of HIIT (90% HR_max_) significantly increased mitochondrial capacity evidenced by an increase in citrate synthase activity and raised content of electron transport chain complexes [12]. In contrast, 16 weeks of interval walking (70% $\overset{.}{V}{\mathrm{O}}_{2\mathrm{peak}}$) only elicited changes in citrate synthase mRNA expression but not in citrate synthase activity. However, as stated above, this could be due to the lower intensity of intervals used in this study compared with traditionally adopted HIIT protocols and because muscle biopsies were obtained 5–6 days post-training [14].

Peroxisome proliferator-activated receptor, gamma, coactivator 1, alpha (PGC-1α) regulates muscle mitochondrial biogenesis [18]. Following HIIT, increases in nuclear PGC-1α levels have been observed [19], as well as increases in total PGC-1α vastus lateralis content in biopsy samples. Similar changes were not observed following energy matched MICT [20]. It has been proposed that the fluctuations in ATP turnover during interval training, which differs from the usual steady state conditions of ATP production, could activate signalling pathways that lead to this increase in PGC-1α following HIIT [21].

#### Sarcoplasmic reticulum

Sarcoplasmic reticulum Ca^2+^ handling plays an important role in muscle fatigue and increased Ca^2+^ reuptake into the sarcoplasmic reticulum has been demonstrated following HIIT, but not MICT in adults with the metabolic syndrome [20]. Increases as large as 50–60% were observed in Ca^2+^ reuptake, significantly improving the work capacity of the muscle and thereby contributing to improvements in fitness following HIIT. This study indicates that HIIT provides a more potent stimulus compared with MICT to induce skeletal muscle adaptations. However, definite conclusions cannot be drawn from one small study, with a total sample size of 32 participants.

## HIIT and glucose control

Key studies on the impact of HIIT on glucose control have been summarised in Table 1. These adopt a range of protocols and cover both the acute and training responses to HIIT.

### The acute response

Few studies have assessed the acute response to HIIT in patients with metabolic disorders and those that have are summarised in Table 1. Relative to no exercise, a single session of HIIT reduces same-day postprandial area under the glucose curve in those with impaired fasting glucose [22] or type 2 diabetes [23, 24]. Similarly, HIIT is associated with reduced time of glucose being ≥10 mmol/L [23, 24]. However, studies assessing the effect of HIIT on mean 24 h glucose levels have been less consistent, with some indicating no effect [22, 23, 25], and one showing a reduction but only when the exercise was performed in a fasted state [24]. When compared with energy matched MICT, HIIT tended to be slightly superior [22, 25, 26] (see Table 1). As measurements were not reported much past 24 hours post exercise, the duration of these effects is uncertain. Earlier work relying on changes in fasting glucose to assess impact, suggests a measurable effect may last as long as 72 h post HIIT, but for a shorter period following MICT [26].

Since postprandial glucose excursions are strong predictors of cardiovascular disease [27], which may be due to possible inductions of oxidative stress and micro/macrovascular damage [28], the above findings are of clinical relevance. Unfortunately, the available research does not provide clarity on dose–response in terms of intensity, duration or total energy expenditure. Further head-to-head comparisons of different protocols are required. However, it is worth noting that a protocol of 1 min intervals with 1 min recovery, repeated ten times (a modest time investment), improves acute glucose control [22, 23].

The transient nature of changes in glucose metabolism in response to exercise is well documented. Insulin-independent glucose disposal is increased during and for approximately 60 min post exercise [29]. Insulin-dependent glucose disposal increases for several hours to a few days following exercise [29, 30]. These effects are localised to contracting muscle [29], thus exercise involving a larger muscle mass is preferable. Higher-intensity exercise has been shown to recruit a larger proportion of muscle fibres compared with moderate-intensity exercise [31], which may explain greater improvements in glucose regulation following HIIT. In light of these acute adaptations, patients should be recommend not to have more than two exercise-free days, in accordance with guidelines [32].

### The training response

Of the studies assessing the effects of longer-term HIIT (≥2 weeks), some report reduced fasting glucose [11, 20, 26, 33, 34], while others report no change [35–39] (Table 1). Where reductions in fasting glucose are observed, they appear to be similar to those seen following MICT [7]. Fasting glucose is predominantly a marker of hepatic insulin sensitivity. After just 1 week of a diet very low in energy (very low calorie diet; 2510 kJ/day [600 kcal/day]), liver fat content decreased by 30%, hepatic insulin sensitivity significantly improved and fasting glucose fell by 35% in adults with type 2 diabetes [40]. The reduction in fasting glucose following participation in HIIT is generally smaller [11, 20, 26, 34, 41], (≤14%, see Table 1), suggesting that exercise (whether HIIT or MICT), lacks potency for improving hepatic insulin sensitivity, when compared with consumption of a very low calorie diet. This is most likely because exercise elicits a smaller energy deficit than that achieved with a modest change in eating behaviour. For example, to achieve an energy deficit similar to that achieved by reducing energy intake by the equivalent of the energy in a blueberry muffin (∼1891 kJ [452 kcal]), a 68 kg female would need to run approximately 38 min at a pace of 9.7 km/h [42]. We did, however, show that HIIT was able to significantly reduce liver fat and, thereby, fasting glucose in some type 2 diabetes individuals [38], but the average reduction in liver fat did not result in a significant reduction in fasting glucose levels. Whether an increase in the duration of the HIIT intervention (i.e. >12 weeks) would achieve a reduction in fasting glucose levels in this cohort is yet to be determined.

HIIT has been shown to improve peripheral insulin sensitivity in those with impaired metabolic control. The molecular adaptations to HIIT described above, including raised GLUT-4 content, increased aerobic enzyme capacity and mitochondrial biogenesis, have all been associated with improved peripheral insulin sensitivity [13, 43]. Studies assessing the metabolic impact of HIIT in those with common metabolic diseases have found no change in HOMA-IR [37–39, 44, 45], whereas others have shown an approximate 20% improvement compared with a control group [20, 33, 34, 41] (Table 1). When compared with MICT, HIIT seems to have a small but significant benefit on insulin resistance [7].

HIIT can also decrease HbA_1c_ [34, 38, 41, 44], yet some studies have reported no change [11, 36, 39, 46] (Table 1). Although there have been a number of studies published since, a meta-analysis found that a 0.47% absolute reduction in HbA_1c_ is observed with HIIT in adults with common metabolic diseases, compared with controls [7]. This is slightly lower than the 0.6% absolute HbA_1c_ reduction observed following aerobic and resistance exercise in type 2 diabetes [47]. Both HIIT and other forms of exercise compare well with improvements achieved through metformin [48] and are likely to have clinical benefits, since a 1% absolute rise in HbA_1c_ leads to a 21% increased risk of diabetes related death, a 14% increased risk of myocardial infarction and a 37% increased risk of myocardial infarction [47].

Other indicators of glucose control, such as 2 h glucose following an oral glucose challenge and glucose AUC are similarly inconsistent across studies (see Table 1). Several explanations for the reported inconsistencies across studies include differences in study populations, exercise protocols and the degree of volunteer supervision during exercise. However, the most plausible explanation is the variation in time of post-intervention measures relative to the last bout of exercise. Studies reporting both the acute and cumulative effect of HIIT have consistently shown that changes in indicators of glucose control last between 24 and 72 h post exercise [25, 26, 33, 36] (Table 1). Only one study has demonstrated a longer-term adaptation, in which fasting insulin was reduced 96–120 h post exercise [36]. Greater inter-study consistency in the timing of post exercise assessments is warranted in the future; continuous glucose monitoring for at least 72 h post exercise and HbA_1c_ assessments may also allow us to gauge benefit better.

Collectively, the improvements in glucose control following HIIT are clinically relevant but do not surpass those seen following the traditionally used MICT with regards to fasting glucose, HbA_1c_ and fasting insulin [7]. HIIT does seem to lead to greater improvements in peripheral insulin sensitivity [7], but overall the use of HIIT for improving glycaemic outcomes should not be over-emphasised compared with other forms of exercise training.

## HIIT and cardiovascular health

Cardiovascular complications are the leading cause of mortality in those with common metabolic diseases [49, 50]. The interval design of HIIT to include rest periods enables patients to accumulate time at higher exercise intensities, thereby challenging the cardiovascular system. Limited evidence indicates that HIIT provides a stronger stimulus than MICT for eliciting myocardial improvements. Alongside the beneficial impact of HIIT on vascular and cardiorespiratory fitness, this suggests that the cardiovascular benefit of HIIT outweighs the metabolic benefit.

### Cardiac adaptations: molecular mechanisms

Because of the difficulty of obtaining human myocardial tissue, most evidence for the molecular adaptations to high-intensity exercise comes from experimental rodent models, the hearts of which bear similarities to human hearts and mimic the human cardiac response to exercise training [51, 52]. The *db/db* mouse model provides a good representation of the human heart in diabetic patients. Following 13 weeks of HIIT, contractility and Ca^2+^ handling were restored to normal levels as a result of raised transverse tubule (T-tubule) density, sarcoplasmic reticulum synchrony of Ca^2+^ release and sarcoplasmic reticulum Ca^2+^-ATPase (SERCA2a; Ca^2+^ transporter) activity [53]. These adaptations occurred despite no improvement in glucose or insulin levels, demonstrating the direct impact of HIIT upon the myocardium. Similar adaptations have been observed in heart failure and healthy rodent models [54, 55], with greater changes occurring following high-intensity exercise (85–90% maximal oxygen consumption [$\overset{.}{V}{\mathrm{O}}_{2 \max }$]) compared with moderate-intensity exercise (65–70% $\overset{.}{V}{\mathrm{O}}_{2 \max }$) [55].

Exercise also activates the phosphoinositol-3 kinase/Akt/mammalian target of rapamycin (mTOR) signal transduction pathway that leads to higher ribosomal biogenesis and protein synthesis, and thus induces physiological hypertrophy to a greater extent following high- (85–90% $\overset{.}{V}{\mathrm{O}}_{2 \max }$) vs moderate- (65–70% $\overset{.}{V}{\mathrm{O}}_{2 \max }$) intensity exercise [52, 55]. The exercise-induced pathways activated in disease models may differ [54], but both healthy and disease rodent models indicate that exercise stimulates important transcriptional, translational and post-translational regulatory mechanisms that lead to structural remodelling of cardiac tissue and, thereby, improved strength of cardiac contractions [52].

### Cardiac structure

Adults with common metabolic diseases display left ventricular concentric remodelling, which represents a reduction in end-diastolic volume (EDV) and is also known as pathological hypertrophy [56, 57]. This reduction in EDV occurs in response to stress signals and is reflective of a build-up of collagen in the myocardium [58]. HIIT, on the other hand, has been shown to induce physiological hypertrophy [38], increasing left ventricular wall mass and EDV by means of a physiological response to growth signals [58]. The number of studies investigating cardiac structure following HIIT is small; our group showed an 8 ml increase in EDV following 12 weeks of HIIT in type 2 diabetes patients [38], but no improvements in NAFLD patients [39]. Both of these studies compared HIIT with a non-exercise control, rather than MICT. That being said, HIIT has been shown to be superior to energy matched MICT in eliciting structural remodelling in those with hypertension [59] and heart failure [60].

### Cardiac function

#### Systolic function

Stroke volume and ejection fraction, two measures of the contractile capabilities of the heart, are reduced in those with metabolic disease [57]. Twelve weeks of HIIT induces systolic improvements in adults with type 2 diabetes [38, 44], hypertension [59] and heart failure [60]. Following 12 weeks of HIIT in heart failure patients, Wisløff et al [60] demonstrated a 35% and 17% relative increase in ejection fraction and stroke volume, respectively, but no change in these variables following energy matched MICT. These improvements are equal to those seen with commonly used prescription medications, such as ACE inhibitors or beta blockers [61]. Twelve weeks of HIIT in hypertensive patients improved early events in systole, which correlate to contractility and are load independent [59]. Furthermore, 12 weeks of HIIT in heart failure patients led to a 22% increase in global contractility [60]. These improvements were not observed following energy matched MICT [60].

Cardiac torsion describes the twisting motion of the heart during contraction and reflects the dominance of epicardial fibres over endocardial fibres. In adults with metabolic disease cardiac torsion is raised [62], reflecting damage to endocardial fibres. Interestingly, we observed reductions in cardiac torsion in adults with type 2 diabetes and NAFLD who partook in 12 weeks of HIIT, when compared with controls [38, 39], suggesting a reduction in endocardial damage following HIIT.

#### Diastolic function

Diastolic dysfunction is often reported in those with common metabolic diseases [57, 63]. Impaired early filling of the left ventricle is indicative of stiffer, damaged myocardial fibres that are less compliant during relaxation; yet evidence suggests that HIIT has the capacity to target these abnormalities. Two studies have demonstrated significant improvements in early filling rates following 12 weeks of HIIT in adults with type 2 diabetes [38, 44], which were sustained 1 year later [44]. Similar diastolic improvements were also observed in adults with NAFLD [39]. These HIIT-induced elevations in early filling rate have been demonstrated to be as large as 49%. In contrast, 12 weeks of MICT fails to have any impact upon diastolic variables [44, 59, 60]. These data suggest that exercise intensity is an important characteristic for inducing diastolic improvements. Diastolic dysfunction is an independent predictor of mortality [64], therefore any improvements in function are likely to be clinically significant.

### Vascular function

Endothelial dysfunction is associated with metabolic disease [65] and considered one of the earliest pathophysiological processes in the progression to atherosclerosis. Flow mediated dilation (FMD) is a measure of endothelial dysfunction and is regulated by NO availability. In those with common metabolic disease, HIIT has been shown to be superior [44] or similar [35] to MICT for improving FMD. Although not limited to common metabolic diseases, a meta-analysis of 182 participants demonstrated twice the improvement in FMD following HIIT, compared with MICT [66]. This is most likely due to the greater shear stress experienced during higher-intensity exercise, since shear stress is the main stimuli for increasing NO availability in the endothelium [59]. Consequently, improved FMD results in greater perfusion and oxygen supply to peripheral tissue.

Findings with respect to the effect of HIIT on blood pressure in individuals with common metabolic diseases have been inconsistent; some studies demonstrate improvements [11, 20, 35, 45], whereas some show no change [36, 38, 39, 44] in blood pressure, despite positive cardiac remodelling [38]. Exercise guidelines for the treatment of hypertension advise low- to moderate-intensity exercise [67], but these findings suggest further work is required to better define the role of HIIT in hypertension therapy.

## Skeletal muscle and cardiac adaptations combine to improve $\overset{.}{\boldsymbol{V}}{\mathbf{O}}_{2\mathbf{peak}}$ following HIIT

It could be argued that the most important outcome following HIIT is cardiorespiratory fitness, as measured by $\overset{.}{V}{\mathrm{O}}_{2\mathrm{peak}}$. Large prospective studies have demonstrated fitness to be more important than established risk factors for mortality [68], and low $\overset{.}{V}{\mathrm{O}}_{2\mathrm{peak}}$ is independently associated with incident type 2 diabetes [69]. While the exercise-induced increase in $\overset{.}{V}{\mathrm{O}}_{2\mathrm{peak}}$ has never been directly linked to mortality, large scale studies indicate that improvements in fitness over time leads to significant reductions in mortality risk [70, 71].

$\overset{.}{V}{\mathrm{O}}_{2\mathrm{peak}}$ is the gold standard measure of fitness and a strong indicator of how well the cardiac, pulmonary, vascular and peripheral systems are working together. A number of meta-analyses have demonstrated the substantial benefits of HIIT for $\overset{.}{V}{\mathrm{O}}_{2\mathrm{peak}}$ and its superiority in comparison to MICT in healthy [72], coronary artery disease [73] and cardiometabolic disease patients [8]. In those with elevated cardiometabolic risk, the increase in $\overset{.}{V}{\mathrm{O}}_{2\mathrm{peak}}$ with HIIT (19.4%) was almost twice that of MICT (10.3%) [8]. On average, $\overset{.}{V}{\mathrm{O}}_{2\mathrm{peak}}$ increases by 5.4 ml kg^−1^ min^−1^ following HIIT, and even a smaller improvement of 3.5 ml kg^−1^ min^−1^ has been predicted to improve survival by 10–25% [74].

Figure 2 provides an overview of the skeletal muscle and cardiac adaptations that are likely to contribute to the improvements in $\overset{.}{V}{\mathrm{O}}_{2\mathrm{peak}}$ observed with HIIT. As demonstrated in the figure, HIIT improves the capacity of both aspects of the oxygen supply and demand chain, but it is the cardiovascular adaptations in response to HIIT that are more likely to contribute to these $\overset{.}{V}{\mathrm{O}}_{2\mathrm{peak}}$ improvements [75].

## HIIT and weight loss

HIIT induces moderate weight loss (0.5–4 kg reduction) in adults with common metabolic diseases [11, 20, 34, 36, 41, 46]. When compared with MICT, however, HIIT provides no additional benefit as an exercise therapy for weight loss [7]. The ability of HIIT to induce reductions in body weight should therefore not be overstated in those with common metabolic diseases.

Although weight loss is strongly associated with reduced metabolic complications [76], it does not reflect changes in body composition; HIIT generally reduces whole body fat mass by 1–3 kg, even when body weight remains stable [14, 35, 36, 41, 46, 77, 78]. Significant reductions in visceral and hepatic fat have also been shown with HIIT [14, 38, 39]. These findings are important since these fat depots increase cardiovascular disease risk [79], and metabolic dysfunction [40, 80]. Three possible mechanisms for HIIT-induced fat loss have been suggested:

1. increased mitochondrial density and capacity following HIIT leading to increased fat oxidation [81]
2. large elevations in catecholamines, which have been shown to drive lipolysis [82], especially in the abdominal tissue where there are significantly more β-adrenergic receptors, compared with subcutaneous fat [83]
3. appetite suppression: energy intake the day after HIIT was ∼1255 kJ (300 kcal) lower than after MICT, and ∼2510 kJ (600 kcal) lower than after rest [84]

It remains unclear whether HIIT is superior to MICT for fat loss, with some studies supporting this notion [14, 36, 46] and some not [34, 35, 41, 45, 78]. To date, evidence to support HIIT over other types of exercise for the management of body fat levels is unfounded, but there is enough proof to suggest that HIIT can induce positive changes in body composition in adults with common metabolic diseases.

A summary of the effects of HIIT can be found in the text box ‘Summary of HIIT’.
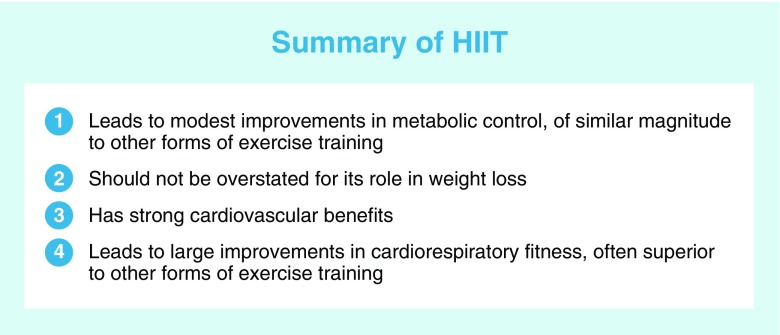


## Is HIIT safe?

Given the strong cardiovascular-focused physiological response to HIIT, it is appropriate to define the safety of high-intensity activity in those at elevated cardiometabolic risk. The acute cardiac response to HIIT has been assessed in a few studies. In patients with coronary heart disease, no contraindications to HIIT were observed and undesirable changes, such as ST-segment depression, recovered during interval recovery periods [85, 86]. Also, in patients with chronic heart failure, cardiac stress (as assessed by rate pressure product) stayed within acceptable values [87]. Furthermore, the studies mentioned above did not report any serious adverse events with HIIT.

The largest available dataset assessing the safety of HIIT was derived from a clinical audit of 4846 cardiac rehabilitation patients. It identified two non-fatal cardiac arrests in 46,364 h of supervised HIIT, and one fatal cardiac arrest in 129,456 h of supervised MICT [88]. Although the low frequency of events makes the comparison between the two exercise modalities inconclusive, it also highlights that the risk of either approach is low. It is important to note that all patients were referred to cardiac rehabilitation by their general practitioner or hospital cardiologist and underwent a full medical screening and cardiopulmonary exercise test prior to taking part, to rule out recurrent ischaemia or chest pain during exercise.

The risk of sudden cardiac death and acute myocardial infarction is increased following vigorous activity in susceptible individuals, including those with structural heart disease and congenital complications [89]. The American College of Sports Medicine and the American Heart Association provide guidelines for identifying high risk patients and carrying out pre-exercise screening in such individuals [6, 89]. According to these guidelines those with common metabolic diseases are automatically considered ‘high risk’. On the whole, however, mounting clinical evidence supports HIIT as a safe therapy for the majority of individuals with elevated cardiometabolic risk.

## Tolerability of HIIT in patients

The trials published to date illustrate the tolerability of HIIT among diverse clinical populations and with varying study durations (see Table 1). Although large-scale trials are lacking, attempts have been made to assess the palatability of HIIT in previously sedentary populations. A group of obese women, some with type 2 diabetes, were noted to prefer a HIIT approach to MICT [90], as did volunteers with coronary heart disease [85]. Within HIIT protocols, enjoyment decreases with increasing interval length [91]. Specifically, intervals of 30 or 60 s resulted in greater enjoyment than 120 s intervals.

Good adherence to free-living, non-supervised HIIT (<3 months) has been demonstrated in those with type 2 diabetes [38], NAFLD [39] and those with either impaired glucose tolerance or impaired fasting glucose [92]. Good adherence was also observed with interval walking (3 min alternative fast and slow walking) in a free-living environment over 4 months in patients with type 2 diabetes [36] and over 22 months in older adults [93]. Additional longer-term studies are required but, nonetheless, these results indicate good adherence and tolerability to independent HIIT exercise.

## Considerations when prescribing HIIT

Beyond the plethora of specific protocols to choose from, the way in which HIIT protocols are often described is, in itself, a barrier to clinical implementation. The text box, ‘Recommendations for HIIT prescription’ provides a summary of our recommendations for prescribing HIIT in a clinical setting.
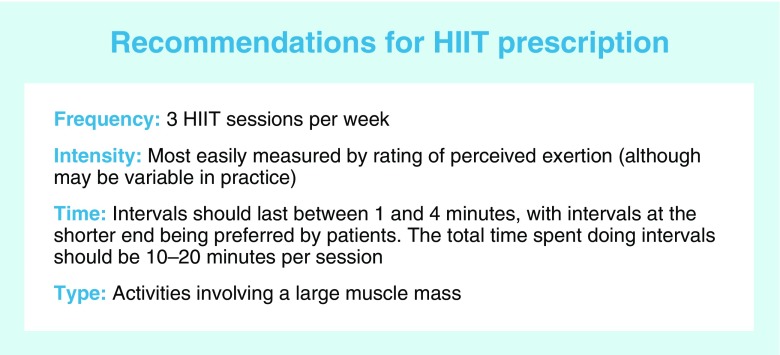


In research, HIIT is most commonly carried out as three sessions per week (Table 1). Such a frequency is consistent with the probable duration of the metabolic effects observed. As previously mentioned, the duration of intervals also varies from 1–4 min (see Table 1). Since longer intervals have not been conclusively shown to yield better clinical outcomes but have been shown to reduce enjoyment [91], it makes sense to start at the shorter end of the range. The accumulated time at high-intensity during HIIT has varied from 10–20 min; starting at the lower end of this range allows for greater progression. Likewise, ratios of interval:recovery time also vary, with a 1:1 ratio offering a simple starting point. Last, keeping the intensity of the recovery period to a minimum is likely to increase enjoyment, at least initially.

Intensity is commonly measured using HR_max_. At first glance, heart rate appears like a feasible option given the relative ubiquity of heart rate monitors. However, heart rate rises across intervals, as shown in Fig. 1. In our research, we have adopted a very practical approach, using the Rate of Perceived Exertion (RPE) 6–20 Borg scale as a guide of intensity; as previously reported, participants were asked to work at a 16–17 on the scale (or ‘very hard’) during each interval (Fig. 1) [38, 39]. RPE is an accurate predictor of exercise intensity in diabetes patients [94], however, like heart rate, it does have its limitations. Agreement between RPE and more objective measures of intensity is known to suffer both inter- and intra-individual variation. For example, when RPE was assessed during a set workload protocol, RPE increased from the first to the last interval [12]. Thus, using RPE trades some objectivity, but the benefit is a great deal of practicality.

Exercise selection is ultimately limited by what is available to the patient. Since peripheral metabolic adaptions are limited to the muscles undergoing forceful contractions during exercise [29], it is preferable to choose activities involving a large muscle mass.

## What’s next for HIIT?

HIIT leads to modest improvements in metabolic control and weight loss. This is in contrast to calorie restriction, which leads to significant weight loss and improvements in metabolic control [40]. Combining HIIT with calorie restriction would accrue the cardiac benefits of HIIT and the weight loss benefits of calorie restriction. Additionally, exercise and calorie restriction together have been shown to improve glucose regulation by two-fold compared with the same amount of weight loss induced by exercise or calorie restriction alone [95]. Thus, there may be additive benefits for metabolic control if HIIT was used adjunct with energy restriction.

The myriad of different HIIT protocols adopted in the literature needs to be addressed. A standardised and consistent approach for prescribing HIIT protocols is missing, making it difficult to detect dose–response effects and the thresholds necessary to elicit desired changes. Most clinical HIIT studies have been short term (<4 months, see Table 1) and performed in a laboratory setting. The feasibility, acceptability and efficacy of longer-term HIIT in a real world setting requires investigation before it can be accepted as an alternative therapy for those with elevated cardiometabolic risk.

## Conclusion

In circumstances where HIIT is not feasible, considered potentially unsafe or not well tolerated by an individual , MICT is effective at eliciting important health benefits. However, throughout this review we have shown that, provided unstable cardiovascular disease is excluded, HIIT appears to have a good safety profile and is well tolerated. Compared with other forms of exercise training, the use of HIIT for improving metabolic control and inducing weight loss should not be overstated. However, there are strong positive cardiovascular adaptations to HIIT that confer benefit to a population at risk of cardiac complications and therein lies the importance of HIIT for metabolic disease management. For optimal clinical benefit (improved glycaemic control *and* cardiovascular function), the value of HIIT appears likely to be adjunct to energy restriction, allowing HIIT to certainly make a hit.

## Abbreviations

EDV: End-diastolic volume
FMD: Flow mediated dilation
HIIT: High-intensity interval training
HR_max_: Maximum heart rate
MICT: Moderate-intensity continuous training
NAFLD: Non-alcoholic fatty liver disease
PGC-1α: Peroxisome proliferator-activated receptor gamma, coactivator 1, alpha
RPE: Rate of perceived exertion
$\overset{.}{V}{\mathrm{O}}_{2 \max }$: Maximal oxygen consumption
$\overset{.}{V}{\mathrm{O}}_{2\mathrm{peak}}$: Peak oxygen consumption
